# Hospitals and the Limited Liability Act

**Published:** 1900-06-02

**Authors:** H. Howgrave Graham

**Affiliations:** Secretary. 32, Portland Terrace, Regent's Park, London, N.W.


### EDITOR'S LETTER-BOX.

[Our Correspondents are reminded that prolixity is a great bar to publication, and that brevity of style and conciseness of statement greatly facilitate early insertion.]

### HOSPITALS AND THE LIMITED LIABILITY ACT.

Sir,—Adverting to your correspondent's letter referred to on page 127 of your last issue, it may interest him to know that for reasons such as his letter indicates, this hospital has quite recently been incorporated in the manner mentioned in your note which follows his letter. I may add to the institution which you name (the Home Hospitals Association for Paying Patients) the Denbighshire Infirmary, the Hampstead Hospital, and the Orthopædic Hospital, Dublin, as instances of hospitals incorporated under the Companies' Acts. Incorporation by Royal Charter, to which you refer also, is in some respects a preferable mode of incorporation, but it is much more lengthy in procedure and also more costly. The Home Office fees alone are £120, and the legal expenses are much heavier than in the case of incorporation under the Acts in question, which can be accomplished for about £50. The chief expense is that of an advertisement which must appear (in a rather long form, prescribed by the Board of Trade) twice in at least one paper and which, if the paper is a "London daily" (as in the case of a London hospital it must be), costs some £17. Five guineas (for counsels' fees) must be paid to the Board of Trade, and the fees for registering and stamps at the Companies Office, Somerset House, are £3 10s., if the number of "members" is limited to twenty; while if the number is not limited the registration fee alone is £20. The number of "members" can be increased from (say) twenty at the rate of 5s. per fifty at any time. All the procedure is very simple, and if, as in our case, a kind legal friend drafts the documents, the above figures indicate the whole necessary expenditure, except that of printing the memorandum and articles of association, which is not absolutely imperative, but is very convenient. I believe that all the practical advantages of incorporation are attained under the Companies' Acts, but the possession of a Royal Charter no doubt confers a "dignity" which the larger hospitals may be justified in paying—and waiting—for. In our case time was of importance. If your correspondent will write to me, I shall be pleased to give him any further help.—Yours faithfully, H. Howgrave Graham, Secretary.

32, Portland Terrace, Regent's Park, London, N.W.
May 22nd, 1900.

